# Foraging zebra finches (*Taeniopygia guttata*) are public information users rather than conformists

**DOI:** 10.1098/rsbl.2020.0767

**Published:** 2021-06-23

**Authors:** Edwin J. C. van Leeuwen, Thomas J. H. Morgan, Katharina Riebel

**Affiliations:** ^1^ Behavioral Ecology and Ecophysiology Group, Department of Biology, University of Antwerp Department of Biology, Antwerp 2020, Belgium; ^2^ Centre for Research and Conservation, Royal Zoological Society of Antwerp, K. Astridplein 26, B 2018 Antwerp, Belgium; ^3^ Department for Comparative Cultural Psychology, Max Planck Institute for Evolutionary Anthropology, Deutscher Platz 6, 04103 Leipzig, Germany; ^4^ School of Human Evolution and Social Change and Institute of Human Origins, Arizona State University, 900 South Cady Mall, Tempe 85287, AZ, USA; ^5^ Institute of Biology, University of Leiden, 2333 BE Leiden, The Netherlands

**Keywords:** frequency-dependent learning, zebra finches, conformity, social learning

## Abstract

Social learning enables adaptive information acquisition provided that it is not random but selective. To understand species typical decision-making and to trace the evolutionary origins of social learning, the heuristics social learners use need to be identified. Here, we experimentally tested the nature of majority influence in the zebra finch. Subjects simultaneously observed two demonstrator groups differing in relative and absolute numbers (ratios 1 : 2/2 : 4/3 : 3/1 : 5) foraging from two novel food sources (black and white feeders). We find that demonstrator groups influenced observers' feeder choices (social learning), but that zebra finches did not copy the majority of individuals. Instead, observers were influenced by the foraging activity (pecks) of the demonstrators and in an anti-conformist fashion. These results indicate that zebra finches are not conformist, but are public information users.

## 1. Introduction

Context-related information can optimize activities like foraging, predator defence and reproduction. Attending to the actions of conspecifics (and their consequences) is an effective way to obtain this information, a process referred to as ‘social learning’ [1,2]. For social learning to be adaptive, however, learning should be contingent on situational features and/or guided towards certain characteristics of the conspecific(s) that on average will lead to a better outcome than random or no copying [3,4]. Positive frequency-dependent social learning in the form of *conformity*^1^ has been identified as one potential strategy for acquiring relevant information about a given environment [6,8,9]. Conformity requires that the probability of adopting the majority variant is larger than the relative majority size (i.e. a *disproportionate* tendency to adopt the behaviour of the majority [3]) and stabilizes popular traditions. It can be contrasted with anti-conformity, which still favours majority options, but to a lesser extent, such that traditions are not stable. Because conformity allows learners to aggregate the knowledge of many individuals [10,11], it can increase the accuracy of decision-making, and, in turn, fitness. As such, conformity is considered to be likely to evolve as a learning strategy in social animals [6,8].

Conformity has been reported in humans (e.g. [7,12]), fruit flies (*Drosophila melanogaster*) [13] and sticklebacks (*Pungitius pungitius*)^2^ [14], with additional indirect evidence coming from field experiments with great tits (*Parus major*) [15] and vervet monkeys (*Chlorocebus aethiops*) [16]. Yet, in several cases (e.g. [14–16]), while the observations were in line with conformity, alternative learning biases could not be ruled out [17–19]. To identify conformist behaviour, and improve the resolution of its phylogenetic analysis, paradigms that account for such alternative explanations are warranted.

In this study, we tested for majority influences in zebra finches (*Taeniopygia guttata*): a group-living and socially foraging bird species observed to engage in social learning in a variety of paradigms (e.g. [20–25]). Using an experimental observer–demonstrator approach [23], we manipulated the number of demonstrators at each of two feeders and investigated whether observer zebra finches preferentially copied the majority. Conformity would be concluded from a sigmoidal relationship between the majority size and an observer's propensity to adopt the majority behaviour [6,7]. However, to overcome the interpretation predicament identified in other conformity studies [17–19], we also tested for alternative strategies, including anti-conformity and ‘public information use’, whereby observers attend to the feeding activity of demonstrators (as opposed to their number), using it as a cue of resource quality [26]. Sticklebacks, for instance, have been observed to preferentially rely on public information over majority size [27]. Thus, to test for public information use, we recorded the pecks of demonstrators at each feeder, and we additionally recorded the visits of demonstrators to each feeder. Our goal, given that social learning appears taxonomically widespread, is to explore the precise form it takes in zebra finches.

## 2. Material and methods

### (a) Subjects and housing

Subjects were 93 domesticated zebra finches (approx. 2 years, range 1–4) from an indoor colony at Leiden University. Sixty-nine (35f/34m) birds started as naive observers that after testing were added one by one to a set of 24 birds (12f/12m) that had been the seeding pool of demonstrators (see electronic supplementary material). Prior to testing, housing was standardized by moving all subjects into the same room into unisex group cages (40 × 80 × 40 cm, housing—two to four birds each, light : dark schedule 13.5 : 10.5 h, 20–22°C). Birds were provided with drinking water, grit, cuttlebone and tropical seed mixture *ad libitum*, supplemented twice a week with germinated seeds, greens and egg food.

### (b) Experimental set-up and procedure

**Figure 1.**
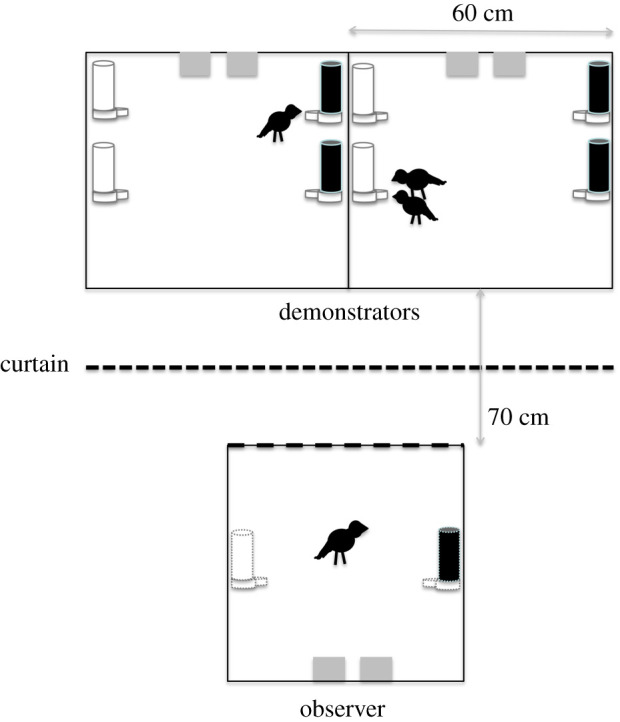
Experimental set-up: observer and demonstrator cages contained sand and grit bedding and were equipped with two water dispensers (grey squares) and three perches (not shown). Black and white feeders were added at the indicated spots during the demonstration (for demonstrators) and testing (for observer) phases. The feeders in the observer cage (depicted with dashed lines) were absent during demonstrations and only added after the demonstration was over and the curtain (dashed line between the cages) had been shut again. The location placement of the black and white feeders was counterbalanced across trials. The cages stood 70 cm apart and could be visually separated by a dark textile curtain that was suspended from above and hung midway between the two cages.

The experiment used an observer–demonstrator paradigm slightly modified from a previously validated paradigm in Leiden ([23]; for additional details and validation see electronic supplementary material). A single cage (for the observer) was positioned such that it faced two cages (for the two demonstrator groups) across an aisle of 70 cm (figure 1). Each demonstrator cage contained two black and two white feeders—only one colour provided access to seeds, while feeders of the other colour had the dispense mechanism blocked.

Subjects were assigned as observers following a testing schedule that balanced the demonstrator majority : minority combinations (1 : 2; 2 : 4, 3 : 3 : 1 : 5), sex (birds in a trial were either all male or all female), colour and position of the rewarded feeder (black or white, left or right demonstrator cage). All observer birds were naive to the experiment.

Trials started by moving a single observer and two demonstrator groups into the experimental cages (table 1). At this stage, the curtain was shut, and the observer cage only contained water but no food dispensers. During the next 30 min—invisible for the observer—the demonstrator birds could feed *ad libitum* from the open (either black or white) feeders. After 30 min all feeders were removed. One hour later, the feeders were returned to the demonstrator cages and the curtain was opened, where the observer could see the demonstrator birds eating for another 30 min. Then the curtain was closed again, and now the observer received two feeders, one black and one white, identical to those used in the demonstrator cages. For the observers, both feeders were unblocked and placed equidistant from the centre of the cage against one of the sidewalls.

**Table 1.** Summary of the timeline of a test trial outlined for observer (O) and demonstrator (D) birds in parallel. B, black and W, white feeder.

times (in min from start)	observers O	curtain	demonstrators D^a^ (large and small group)
0–30	no food, can hear but not see D's	closed	experiencing two B&W feeders, one colour blocked
30–90	no food	closed	feeders removed
90–120	no food, observing D's	open	feeders returned
120–150	test: B&W feeder	closed	feeders remained
151–	repeat previous step if bird had not been visiting feeders yet, otherwise bird back to home cage, cage cleaning	closed	stay on for another 30 min with observer or, back to home cages, cage cleaning

^a^There were two demonstrator groups differing in group size, the larger always being referred to as ‘majority’. Majority : minority combinations of the demonstrator groups were 1 : 2; 2 : 4; 3 : 3 and 1 : 5.

All demonstrator and observer behaviours were filmed and projected outside the room onto a monitor, where they were scored for visits and pecks by a single experimenter who—owing to the group sizes being visible—was not blinded to the procedure (for more details, see electronic supplementary material). If an observing bird had not yet started visiting one of the feeders within 30 min after receiving the feeders, trials continued for another 30 min (*N* = 7); if these birds still had not eaten by then the trial was aborted and not tested again (*N* = 2). One trial had to be excluded for technical reasons. Consequently, 66 (34f/32m) out of 69 birds were included in the analysis. Only same-sex observer–demonstrator combinations were used to avoid birds engaging in courtship behaviour.

### (c) Statistical analysis

The analysis changed following reviewer feedback, for full history see the electronic supplementary material. Data [28] were analysed with Bayesian generalized linear mixed models using Markov chain Monte Carlo methods to generate parameter estimates in the R package rjags [29]. Estimates are based on a minimum of 3000 effective samples drawn from three chains, and convergence was confirmed with the Gelman–Rubin diagnostic (upper C.I. ≤ 1.01).

We modelled whether the observer bird's first peck was to the black feeder (as opposed to the white feeder) as a Bernoulli variable. We focussed on the first peck only as subsequent pecks may be influenced by individual learning while at the feeders [30], and because birds can peck multiple times within a single visit meaning each peck is not an independent event.

Feeder colour (black or white) was counterbalanced across the minority : majority groups and corrected for in the statistical analyses. Furthermore, we controlled for the side of the cage the black feeder was on. The presence and behaviour of the demonstrator birds (the main predictors of interest) were quantified in three ways: the proportion of demonstrator birds that were at the black (as opposed to white) feeder (*birds*), the proportion of visits the demonstrators made that were to the black feeder (*visits*) and the proportion of demonstrator pecks that were at the black feeder (*pecks)*. To test for a conformist (or anti-conformist) response to the demonstrators, we estimated the effect of these variables using the function for conformist transmission developed by Boyd & Richerson [8]. Owing to substantial multicollinearity between the main predictors, particularly *visits* and *pecks*, all three cannot be included in the same analysis (see electronic supplementary material). As such, we conducted the following analysis in which an indicator variable was used to choose between *birds*, *visits* and *pecks* (for analyses including both *birds* and *visits*, and both *birds* and *pecks*, with qualitatively unchanged results, see electronic supplementary material).

observer pecks black first∼Bernoulli(p),

$$\mathrm{logit}(p)=\;\beta_{1}+\beta_{2}\times \mathrm{black}\;\mathrm{position}+\beta_{3}\times \mathrm{social}\;\mathrm{information},$$

social information=X+cX (1−X)(2X−1)−0.5,

$$\beta_{1:3}∼\mathrm{Normal}(0,\mathrm{precision}=0.1),$$

$$X=\{\begin{array}{c} \mathrm{birds},\;m=1\\ \mathrm{visits},\;m=2\\ \mathrm{pecks},\;m=3, \end{array}$$

c∼Normal(0,precision=0.1)

$$\;\;\;\;\;m∼\mathrm{Categorical}\left(\frac{1}{3},\;\frac{1}{3},\;\frac{1}{3}\right).$$

In this case, fixing the value of *m* allows us to test the effects of *birds*, *visits* and *pecks* separately, while the posterior distribution of *m* provides information about the relative predictive power of the three variables. The parameter *c* determines whether or not the response is conformist.

## 3. Results

**Figure 2.**
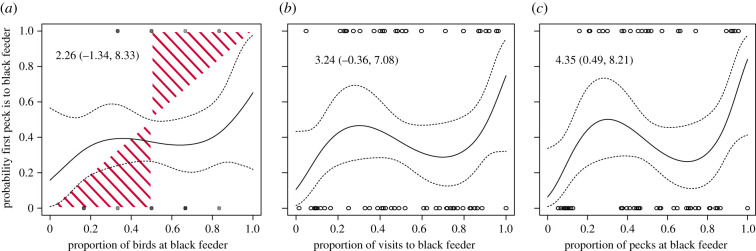
Regression of whether the observer bird's first peck was at the black feeder (*y*-axis) on to (*a*) the proportion of demonstrator birds at the black feeder (the red shaded area corresponds to conformist transmission), (*b*) the proportion of demonstrator visits to the black feeder and (*c*) the proportion of demonstrator pecks at the black feeder (all on *x*-axis). Points show the raw data (semi-transparent in (*a*) as many overlap), while lines show the median estimate (solid) and 95% central credible interval (dashed). Model estimates for coefficients are included in all panels. Note that strong evidence of an effect is only found for pecks, and the effect is anti-conformist (*c*). For versions of this figure that directly show the posterior samples or raw data averages, see the electronic supplementary material.

The observer birds showed a slight preference for the white over the black feeder, but no preference for a particular side of the cage. The separate analyses of *birds*, *visits* and *pecks* found little evidence that observers were influenced by the proportion of birds at each feeder (figure 2*a*), weak evidence that they were influenced by the proportion of visits to each feeder (figure 2*b*), but strong evidence that they were influenced by the proportion of pecks at each feeder (figure 2*c*). The minimal response to the number of birds (figure 2*a*) does not match the sigmoidal pattern typical of conformity [3,6]; however, the response to pecks exhibited an anti-conformist pattern (figure 2*c*). The indicator variable favoured pecks (weight = 0.67) over birds (0.10) or visits (0.23). For quantitative model estimates, see table 2.

**Table 2.** Summary of model results. Values are shown for each of the three predictor variables.

variable	interpretation	estimate (median and 95% CCI)
*β*_1_	baseline preference for black feeder on logit scale	birds: −0.51 [−1.04, −0.01]
		visits: −0.52 [−1.05, −0.01]
		pecks: −0.51 [−1.06, 0.01]
*β*_2_	preference for visiting feeder on a particular side on logit scale	birds: 0.24 [−0.77, 1.26]
		visits: 0.28 [−0.76, 1.35]
		pecks: 0.28 [−0.77, 1.36]
*β*_3_	change in value of linear predictor per unit increase in proportion of birds, visits or pecks at black feeder	birds: 2.26 [−1.34, 8.33]
		visits: 3.24 [−0.36, 7.08]
		pecks: 4.35 [0.49, 8.21]
*c*	shape of response to birds, visits or pecks (e.g. conformist, anti-conformist)	birds: −2.42 [−5.25, 3.61]
		visits: −3.74 [−6.29, 1.84]
		pecks: −3.79 [−5.92, −0.17]

## 4. Discussion

In this study, we experimentally tested for majority influences in zebra finches. We found little evidence for an effect of the proportion of demonstrators exhibiting a behaviour (i.e. choosing one feeder colour over the other) on the probability that an observer adopts that same behaviour, and no evidence for a conformist response [3]. Instead, zebra finches responded to demonstrator feeding activity (i.e. pecks at the feeder). This is consistent with *public information use*: extracting information inadvertently produced by others to assess resource quality [25,26,31]. However, the shape of the response was anti-conformist: when the pecks overwhelmingly favoured one feeder, observers followed suit, but when the information was more mixed, observers favoured the less popular feeder. Further work could test whether zebra finches conform in different contexts (e.g. mate choice [13]) or when public information is unavailable (e.g. adaptations of [7]) to assess whether conformity is part of the decision-making process of zebra finches at all.

The anti-conformist response to observed pecks is unexpected and requires explanation. One hypothesis is that when pecks were plentiful at both feeders, observers chose the minority feeder to avoid competition, but when pecks were heavily biased toward one feeder observers concluded that that feeder was far richer than the other. Similar results were observed in sticklebacks, where the response of observers to demonstrated feeding behaviour (as well as the number of demonstrators) was much more pronounced when the demonstration was heavily biased than when it was more mixed [14], and an anti-conformist response to the number of demonstrators has also been documented in young children [32].

Our results are broadly consistent with other studies that found evidence for social learning in zebra finches [20–24,33,34]. However, these findings have not been consistent, raising concerns about their validity [35]. While it is possible that published positive results are false positives, there is also evidence that zebra finch social learning is context dependent [23], meaning that design differences across studies could prompt or suppress this behaviour. Our findings may partly explain these discrepancies as most studies do not account for feeding behaviour (as opposed to the number of individuals) or an anti-conformist response. Additional work, including validation and replication, is required to establish results with certainty (also see [35]).

The role of conformity in decision-making across species has gained much traction (e.g. [13–16,36]) because it offers a means to stabilize cultural traditions, and while cultural traditions seem abundant among humans and animals (e.g. [37–40]), their evolutionary origins and behavioural mechanisms are poorly understood. However, despite substantial research efforts, the evidence for conformity in non-human animals is mixed (see [11]), with evidence both in favour (e.g. female fruit flies' mate choice [13], foraging decisions of great tits ([15], yet cf. [5]), and vocal learning in swamp sparrows [41]) and against (oviposition site choice in fruit flies [42], foraging in sticklebacks [14] and dietary preferences in chimpanzees [43]). Here, we find evidence of an *anti*-conformist response to *pecks*, and not a conformist response to individuals. Such a response may erode, rather that sustain, traditions and thereby enable zebra finches to better explore food sources in their environment.

The possibility of anti-conformity (and public information use) may explain why evidence for conformity is mixed. However, other possible reasons include current operational ambiguities of the different forms of majority influence (see [11,44,45]), and differences in the methods used to study these variants (e.g. sigmoidal curve fitting [15] versus counting behavioural adjustments [16]). Moreover, mechanisms other than conformity (e.g. an ‘expert bias’) can result in similar sigmoidal patterns [18,46,47] even though this pattern is typically used to identify conformity [48–51]. Further work, including theory concerning conformity, anti-conformity and public information use, is required to achieve clarity.

Our study sought to understand which of several potential mechanisms guided social learning in the zebra finch, thereby addressing whether animals (including humans) may be biased toward copying the majority of *individuals* or the majority of *instances* (see [17,52,53]). The frequency of individuals and instances (i.e. behavioural actions) may often be correlated, yet, under plausible conditions, can also diverge, begging the question which takes precedence [53]. In the current study, zebra finches responded to the number of pecks, more so than visits or individuals, and we conclude that foraging zebra finches are public information users rather than conformists. However, we also found that the response to pecks was anti-conformist, raising further questions about the form of social learning in zebra finches and other species more broadly. Our findings highlight the diversity of mechanisms that may underlie animals' social learning and so play a role in the evolutionary origins of culture.

## Footnotes

1 Here used *sensu* the cultural evolution, not the social psychology literature [5]. More specifically, we equate conformity with conformist transmission: a disproportionate tendency to copy the majority [3,6,7].

2 See Discussion for an alternative interpretation of these stickleback findings consistent with *anti*-conformity.

## Data accessibility

All data used in this study are available from the Dryad repository: https://doi.org/10.5061/dryad.hmgqnk9fd [28].
